# Populated Places and Conspicuous Consumption: High Population Density Cues Predict Consumers’ Luxury-Linked Brand Attitudes

**DOI:** 10.3389/fpsyg.2021.728903

**Published:** 2021-12-01

**Authors:** Tobias Otterbring, Michał Folwarczny, Lynn K. L. Tan

**Affiliations:** ^1^Department of Management, University of Agder, Kristiansand, Norway; ^2^Institute of Retail Economics, Stockholm, Sweden; ^3^Department of Business Administration, Reykjavik University, Reykjavik, Iceland; ^4^School of Social Sciences, Singapore Management University, Singapore, Singapore

**Keywords:** population density, luxury consumption, conspicuous consumption, status signaling, brand attitudes

## Abstract

Population density has been identified as an ecological factor with considerable behavioral implications. The present research aimed to examine whether the mere perception of more (vs. less) populated places can change consumers’ luxury-linked brand attitudes. To this end, we experimentally manipulated consumers’ perceptions of population density using pictorial exposure to high (vs. low) population density cues. The results revealed a significant interaction between manipulated population density and perceived brand luxury on brand attitudes. Specifically, exposure to high rather than low population density cues resulted in more positive (negative) attitudes toward brands deemed to be more (less) luxurious. These findings support our prediction that high population density cues can shift people’s perceptions in consumption contexts linked to luxury. Our work contributes to the growing stream of literature on population density and suggests that this (geo-) demographic factor can exert important downstream effects on consumer behavior.

## Introduction

In 2020, the value of the personal luxury goods market worldwide was €217 billion (Sabanoglu, 2021). The growth in this market is largely driven by a set of demographic factors, including country characteristics (Guercini and Milanesi, 2017; Boisvert and Ashill, 2018) and an increased share of the population purchasing luxury goods (Silverstein and Fiske, 2003). In the current research, we add to this stream of literature by examining whether population density cues can change consumers’ luxury-linked brand attitudes. While recent research has found that exposure to high (vs. low) population density cues makes consumers more motivated to avoid common brands and opt for originality in identity-relevant product categories (Matherly et al., 2018), we examine the possibility that such findings, at least in part, may have emerged due to consumers’ greater propensity to signal status when they perceive a place as more (vs. less) densely populated. Therefore, we test the key hypothesis that high (vs. low) population density cues induce more favorable (unfavorable) attitudes toward brands perceived to be more (less) luxurious. This prediction seems plausible given that luxurious brands are characterized by uniqueness and identity relevance (Berger and Heath, 2007; Chan et al., 2012; Otterbring et al., 2021b), with consumers being particularly prone to signal status in public (vs. private) settings, such as in most consumption contexts, when there are others around to impress (Griskevicius et al., 2010; Goenka and Thomas, 2020; Wang et al., 2021).

Considering the size of the global luxury goods market, it is crucial for stakeholders to understand whether and how various (geo-) demographic factors, including population density cues, affect consumers’ attitudes toward brands linked to luxury. Our work seeks to contribute to such knowledge, which can have broad implications for marketing, advertising, and public policy. In the remainder of this article, we use the term conspicuous consumption to denote the act of purchasing and publicly displaying luxurious brands to signal status and wealth to others (Veblen, 1899; Griskevicius et al., 2007; Han et al., 2010; Sundie et al., 2011; Otterbring et al., 2018).

## Theoretical Framework

### Why do People Engage in Conspicuous Consumption?

Throughout human history, social status has always been an important goal; simply consider the grandiose, majestic pyramids and palaces built for ancient Egyptian Pharaohs and Emperors of Imperial China during their monarchical rule. Still today, we are repeatedly exposed to “flashy” Ferraris and affluent accessories from brands such as Chanel, Gucci, and Prada. Hence, it is relevant to understand the underlying motives that may guide consumers’ tendencies to flaunt branded objects to signal status and wealth.

Conspicuous consumption frequently involves purchasing luxury items that can be used to saliently “show off” one’s wealth to others (Saad, 2007; Griskevicius et al., 2010; Otterbring, 2018). The demonstration of wealth serves as a proximal goal to engage in conspicuous consumption, but what is the distal and ultimate explanation for this type of behavior? In other words, does conspicuous consumption serve higher-order goals than merely signaling financial assets? According to the Fundamental Motives Framework (Kenrick et al., 2010), conspicuous consumption facilitates the achievement of certain distal life goals, with social status being the most immediate fundamental aspect addressed through this consumption practice.

High social status endows an individual with access to other vital resources, including strengthened social influence and better health, both physically and psychologically (Marmot, 2004; Nelissen and Meijers, 2011; Otterbring, 2021a). Conspicuous consumption—a proxy for high social status—facilitates the attainment of other adaptive goals linked to survival and reproduction (Penn, 2003; Saad, 2007; Miller, 2009; Otterbring et al., 2020), such as attracting mates (Townsend and Levy, 1990; Griskevicius et al., 2007; DeWall and Maner, 2008; Dunn and Searle, 2010). The latter is especially true for men because women, on average, prioritize the financial prospects of a potential mate more than men do (Buss, 1989; Li and Kenrick, 2006; Valentine et al., 2020). Thus, although conspicuous consumption carries not only high financial costs but also social costs in terms of lowered perceptions of warmth, loyalty, and maturity inferences (Griskevicius et al., 2009; Cannon and Rucker, 2019), people engage in this type of consumption because the benefits are deemed to outweigh the costs.

### Population Density and Conspicuous Consumption

Several demographic factors are associated with the tendency to display status conspicuously. For instance, materialistic values—a predictor of conspicuous consumption—increase with age (Jiang et al., 2021). Affluence and ethnicity also predict consumers’ tendencies to engage in conspicuous consumption (Ryabov, 2016), just as their job position (employer vs. employee), educational level, and gender (Karunanayake, 2020). In the present research, we argue that a largely overlooked factor, consumers’ population density perceptions, could also explain some variance in conspicuous consumption.

Population density has been identified as an ecological factor with important behavioral implications (Ellis et al., 2009; Sng et al., 2018). As population density increases, so does competition for limited resources (Ellis et al., 2009). Therefore, population density may indicate that resources are becoming increasingly scarce (cf. Folwarczny et al., 2021a, 2022) and that people need to be more competitive to achieve their goals (Sng et al., 2018; Sng and Ackerman, 2020). Under cues of resource scarcity, people evolved to adopt behaviors that maximize their fundamental need to survive (Ellis et al., 2009; Griskevicius et al., 2013). One such strategy to gain a competitive edge is status signaling (Shenk et al., 2016; Kruger and Kruger, 2018).

Previous research has found that people living in densely populated places are more materialistic than those living in sparsely populated areas. For example, people living in Singapore—a Southeast Asian densely populated urban country—reported having higher materialistic values than their American peers (Li et al., 2011). Higher materialism among Singaporean (vs. American) women also translates into prioritizing earning capacity in a potential mate (Li et al., 2011), suggesting that women raise their expected standards of potential partners and that men need to demonstrate their wealth more through conspicuous consumption to be competitive on the mating market (Kruger and Kruger, 2018). However, just as population density may increase men’s conspicuous consumption, the same purchase pattern could apply to women because people tend to choose partners of similar social status and engage in assortative mating (Buss and Barnes, 1986; Kalmijn, 1994). Thus, women may also magnify their attempts to signal status in densely populated places, given that they, too, engage in conspicuous consumption to address mating-relevant challenges (Wang and Griskevicius, 2014).

Based on the above-mentioned rationale, we sought to experimentally test the key hypothesis that manipulated population density is sufficient to change consumers’ luxury-linked brand attitudes, such that exposure to cues of high (vs. low) population density increases positive (negative) attitudes toward brands perceived as more (less) luxurious. Taken together, our work contributes to the growing body of literature highlighting motivational and attitudinal effects of population density (Ellis et al., 2009; Sng et al., 2018; Sng and Ackerman, 2020), with our findings suggesting that this (geo-) demographic factor can exert important downstream effects on consumer responses and people’s lust for luxury.

## Materials and Methods

We exposed participants to visual cues of either high or low population density (cf. Matherly et al., 2018; Sng et al., 2018; Sng and Ackerman, 2020) to test whether experimentally manipulated perceptions of population density could affect people’s attitudes toward brands deemed to differ on the luxury dimension. Data, analysis code, and other replication materials are available through the Open Science Framework at https://osf.io/bwje2/.

### Participants

A total of 203 Americans were recruited through Prolific (115 women, *M*_age_ = 34.1 years, *SD* = 12.8, and range = 18–78 years). *A priori* power simulations to detect interaction effects are complex and often inaccurate in mixed-effect models without obtaining at least pilot data (Mathieu et al., 2012); hence, we aimed to recruit 100 participants per cell and then perform a *post hoc* power simulation, with the effect size of our focal interaction obtained from the main study. Observed power analysis with the “simr” package for R (Green and MacLeod, 2016) revealed that our sample size was sufficient to achieve the power of 0.98 (95% confidence interval = [0.97, 0.99]) to detect the interaction effects of interest; hence, this sample size was appropriate for testing our focal hypothesis. Formal approval from an institutional review board was neither needed nor sought as local regulations on research involving human subjects did not require such a procedure in the case of research posing minimal risk of harm to participants. Participants approved an informed consent form before taking part in the study.

### Procedure

Participants were randomly assigned to view one of two different slideshows featuring either high or low population density, followed by listing three challenges that came to their minds when thinking about overpopulation or underpopulation. Then, they evaluated 20 brands in terms of their perceived prestige, social status, and exclusivity (0 = *Not at all*; 100 = *Very much*). These items (20 items measuring each of the following: prestige, social status, and exclusivity) were averaged to create a luxury index for each of the 20 brands (α = 0.96). Note that the luxury index was created for all 20 brands separately, corresponding to 20 repeated measures of this variable. Participants also stated their attitudes toward each of these brands on a single-item scale (−100 = *Very negative*; 100 = *Very positive*). We randomized the order of these tasks (expressing attitudes and evaluating brands) to minimize the influence of order effects on the results. Finally, participants provided demographic information, indicated their Prolific IDs, and replied to an attention check.

### Brands

We conducted a pretest to ensure that participants were sufficiently familiar with the brands used in the main study. The brands ranged from casual to luxurious (for details, see https://osf.io/bwje2/). Participants were exposed to logos of brands such as Old Navy, Nike, Chevrolet, Versace, Porsche, and Rolex. 25 US participants recruited through Prolific stated how familiar they were with 28 brands (0 = *Not at all familiar*; 10 = *Very familiar*) and indicated the extent to which they associated the brands with prestige, social status, and exclusivity (0 = *Not at all*; 100 = *Very much*; for details, see https://osf.io/bwje2/). The 20 brands that participants were most familiar with (*M*_familiarity_ = 7.1, range = 5.7–8.6) were selected for inclusion in the main study. This set covered a wide range of brands that differed markedly in perceived prestige (*M*_prestige_ = 65, range = 30–96), status (*M*_status_ = 67, range = 36–96), and exclusivity (*M*_exclusivity_ = 61, range = 27–96).

### Population Density Manipulation

Another pretest aimed to confirm that participants assigned to the high (vs. low) population density condition deemed living spaces as more (vs. less) scarce, even when controlling for potential differences in positive and negative affect (Thompson, 2007; see the Supplementary Material). Sixty-two US participants recruited through Prolific watched a 40-s slideshow consisting of eight slides. In the high-population density condition, participants were exposed to images of places that were full of people. Each slide had a short note describing challenges related to overpopulation. In the low-population density condition, participants were instead exposed to images of similar places that depicted underpopulation (for details, see https://osf.io/bwje2/). To maximize the effectiveness of our manipulation, right after the slideshow, participants were asked to list three challenges that came to their mind when thinking about overpopulation or underpopulation, respectively, (for a similar procedure, see Mittal et al., 2015; Young et al., 2018). Next, participants filled out a 4-item measure (α = 0.92) on perceived space scarcity, which served as a manipulation check. These four items were: (a) “There are too many people living on Earth,” (b) “Cities have too many inhabitants,” (c) “It becomes increasingly difficult to find quiet spaces,” and (d) “Many people compete for living spaces” (1 = *Disagree Strongly*; 7 = *Agree Strongly*), which were combined to form a composite manipulation check index (α = 0.92). An independent samples *t*-test on the manipulation check index revealed that people exposed to high (vs. low) population density cues perceived living spaces as significantly scarcer (*M*_high density_ = 5.35, *SD* = 1.56; *M*_low density_ = 3.65, *SD* = 2.10), *t*(60) = 3.66, *p* < *0.001*, Cohen’s *d* = 0.94.

## Results

Our data were nested because we took 20 repeated measures of attitudes toward brands and 20 repeated measures of the brands’ perceived prestige, status, and exclusivity. Additionally, we expected the potential effect of the manipulation to differ across participants and brands. Thus, we performed a linear mixed-effects analysis on the relationship between the luxury index, experimental condition (both treated as fixed effects), and their interaction using the “lme4” package for R (Bates et al., 2015) with brand attitudes as the dependent variable. As random effects, we used intercepts for participants and brands. We added by-brand random slopes for the effects of the experimental condition (initially, we also added by-participant random slopes for the effects of the experimental condition, but this random slope had to be removed due to convergence issues; however, both models lead to the same conclusions). Significance was estimated with Satterthwaite’s method (Kuznetsova et al., 2017), and visual inspection of residual plots suggested no apparent deviations from homoscedasticity or normality.

**FIGURE 1:**
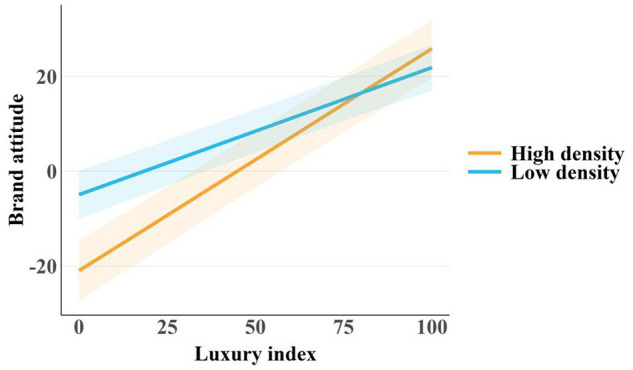
Shaded areas around slopes show 95% confidence intervals. Higher scores on the *Y*-axis correspond to more positive attitudes toward brands, whereas higher scores on the *X*-axis denote brands deemed more luxurious.

The analysis revealed a significant—but not hypothesized—main effect of experimental condition, with participants in the low (vs. high) population density condition generally reporting more positive attitudes toward brands, *b* = 16.04, *SE* = 5.24, *t* = 3.06, *p* = 0.003. Moreover, we found a significant main effect of the luxury index, such that participants had more positive attitudes toward brands they deemed more (vs. less) luxurious, *b* = 0.47, *SE* = 0.05, *t* = 9.70, *p* < 0.001. This effect size, corresponding to a standardized regression coefficient of *β* = 0.29, can be interpreted as a moderate association between the variables (Acock, 2014). More central for the current investigation, the hypothesized interaction effect between experimental condition and the luxury index on participants’ brand attitudes emerged, *b* = −0.20, *SE* = 0.05, *t* = −4.28, *p* < 0.001. This interaction, corresponding to a standardized regression coefficient of *β* = −0.13, is equivalent to a weak association between variables (Acock, 2014). Table 1 shows the unstandardized coefficients along with their confidence intervals. In the high-density condition, the effect of the luxury index on brand attitudes was different than in the low-density condition. Specifically, participants exposed to high population density cues had more positive (negative) attitudes toward brands they deemed as more (less) luxurious than their counterparts exposed to low population density cues, as reflected by a steeper slope on the luxury index-brand attitudes graph; see Figure 1.

**TABLE 1:** Results, unstandardized regression coefficients.

	Brand attitude
Intercept	−20.99**
	[−33.56, −8.43]
Condition (low density)	16.04**
	[5.77, 26.32]
Luxury index	0.47***
	[0.37, 0.56]
Condition (low density) × Luxury index	−0.20***
	[−0.29, −0.11]

**p < 0.01; and ***p < 0.001. Brackets show 95% CIs.

## General Discussion

The present study shows that pictorial exposure to high (vs. low) population density cues results in more favorable attitudes toward brands deemed to be more luxurious. Conversely, for brands rated as less luxurious, exposure to high (vs. low) population density cues led to less favorable attitudes, with this pattern being more pronounced than that for more luxurious brands. These results suggest that exposure to high population density cues makes people more sensitive to signals of status and wealth, potentially because such cues make people more competitive (Sng and Ackerman, 2020).

The larger extremity in negative, as opposed to positive, attitudes among participants shown cues of high population density is consistent with the negativity bias and the “bad is stronger than good”-maxim (Baumeister et al., 2001; Rozin and Royzman, 2001; Otterbring and Shams, 2019). This finding also suggests that brands low in luxury are more detrimental to status signaling than the status gains achieved from highly luxurious brands. Brands low in luxuriousness should take these findings into account when targeting markets characterized by high population density as they may suffer from receiving negative brand attitudes. In contrast, companies offering luxury brands may benefit from targeting densely populated regions (e.g., cities, municipalities, counties, and states) as their brands may be viewed more favorably there. As an anecdotal example, the population density of the United Kingdom is eight times higher than that of the United States (Ritchie, 2019), and the relative share in luxury goods is larger in the United Kingdom than in the United States when adjusting for the different population sizes of these nations (Deloitte, 2019). Given that the United Kingdom and the United States are two of the largest luxury markets worldwide (Chattalas and Shukla, 2015), our findings may have broad and important implications for the promotion and positioning of luxury brands.

Because pictorial exposure to population density cues was sufficient for our effect to occur, this suggests that the mere perception of higher population density may alter consumers’ luxury-linked attitudes. Specifically, luxury brands may strategically use subtle population density cues in their communication campaigns and create the perception of populated places in commercials, advertisements, and on in-store signs located in the vicinity of status-signaling goods to persuade consumers into pricey purchases. However, while the current study focused on the impact of population density cues on brand attitudes, a fruitful avenue for future research is to test whether our findings may be reversed for certain services. For example, reliance on population density cues in promotions of exotic vacations may be counterproductive, as consumers likely prefer the isolation linked to these luxury experiences and value the private nature of such services.

Based on our findings, policy-makers may better understand population density as an environmental factor that could promote preferences for luxury brands. Thus, our results offer potential implications for policy-makers seeking to prevent consumers from incurring high debts through spending frivolously on luxury brands. People who cannot afford luxury brands but reside in highly populated neighborhoods may be particularly vulnerable due to their more favorable attitudes toward luxury-linked brands in such populated places. Indeed, our work suggests that the evolved human striving for status can be triggered by population density cues, possibly urging people to loan money for purchasing pricey possessions. Policy-makers may want to consider implementing regulations or guidelines among retail sectors that are located at highly populated places in areas where people of lower-income brackets live and reflect on whether the availability of luxury brand shops in such areas should be more closely controlled to prevent less affluent people from getting into debt.

## Limitations and Future Research

Our results should be interpreted with caution, given that we did not control for social desirability bias. As participants proceeded to the main task shortly after the manipulation, they may have inferred that the manipulation aimed to alter their subsequent brand attitudes. As such, although web-based, self-administered, and anonymous studies tend to produce more honest responses than studies conducted in classrooms and similar settings (Kreuter et al., 2008), we cannot estimate the degree to which social desirability may have influenced our results. Asking indirect questions reduces social desirability bias in consumer research (Fisher, 1993); hence, researchers may consider less direct measures of brand attitudes or perceived luxuriousness. Future studies could also collect objective, geospatial data on actual population density in areas where participants live instead of solely relying on subjectively perceived population density.

It should be noted that we merely measured brand attitudes rather than behavioral intentions or real, observable behavior. Although research has shown comparable results for attitudes, behavioral intentions, and actual purchase behavior in the context of status-signaling consumption (e.g., Nave et al., 2018; Otterbring et al., 2018), attitudes do not always translate into congruent behavioral responses (Baumeister et al., 2007; Cialdini, 2009; Doliński, 2018; Otterbring, 2021b). Thus, behavioral data are needed before the generalizability of our findings can be concluded with confidence. Ideally, field studies should be conducted to test the replicability and ecological validity of the current results (Machín et al., 2020; Gidlöf et al., 2021; Otterbring et al., 2021a). Relatedly, our study was restricted to participants from Western, Educated, Industrialized, Rich, and Democratic countries (Henrich et al., 2010; Eguren et al., 2021; Folwarczny et al., 2021b). However, many psychological phenomena are culturally contingent, thereby calling for more research across settings, sample types, and cultural contexts (Folwarczny and Otterbring, 2021).

The magnitude of our focal effect was indicative of a weak association between manipulated population density and perceived brand luxuriousness on participants’ brand attitudes. Such a weak effect may be difficult to replicate. Therefore, we recommend testing the replicability and external validity of our results with sufficient statistical power. Nevertheless, the magnitude of our obtained interaction effect was expected, given that related research has found weak associations between population density manipulations and dependent measures such as temporal discounting (Sng et al., 2017). Even large, multinational studies have revealed a significant correlation in the predicted direction only in two-thirds of the cases (Rotella et al., 2021), suggesting that the effects of population densities, either real or manipulated, are small and not always predictable. Thus, more research is needed to estimate the replicability of the current findings. Furthermore, manipulated population densities may affect the life-history strategies that people follow (Sng et al., 2017); therefore, additional studies should optimally control for participants’ life-history strategies to ensure that perceived population density is the main driver of the results reported herein. It is worth noting, however, that even weak effects can have profound practical implications in case they are scalable and hence apply nationally or globally (Funder and Ozer, 2019; Otterbring and Folwarczny, 2022), given that billions of consumers are exposed on a daily basis to cues of high or low population density.

The results of the second pretest showed that our experimental manipulation did not lead to any notable differences in positive affect between population density conditions. However, participants exposed to the high (vs. low) population density condition reported significantly higher levels of negative affect. Although our population density pretest was robust to the inclusion of negative affect as a covariate, future studies should optimally control for such affective influences to rule out this potential confound. Additionally, our 4-item manipulation check referred to a general sense of population density in the world. It is possible that a better way of assessing whether our manipulation influenced participants in a meaningful way would be to ask them about the perceived immediate local population density (e.g., “How difficult is it to find a quiet space in your neighborhood,” or “People in my neighborhood compete for housing”). Ideally, a comprehensively validated scale that measures the extent to which people perceive population density as high or low should be used to ensure that a manipulation works as intended. In addition, researchers should appropriately choose between formative and reflective measurement models when using such an instrument (cf. Fleuren et al., 2018).

Our findings contradict related work on social density (i.e., differing levels of crowding), which has demonstrated that people typically assess *higher* prices and report a *greater* willingness to pay for products presented in less (vs. more) crowded contexts, with this effect being driven by status-motivated individuals’ attempts to associate themselves with people of higher-status (O’Guinn et al., 2015). Future research should address these mixed findings and disentangle under which specific circumstances populated places, crowding cues, and public consumption contexts increase or decrease conspicuous consumption. Finally, future scholarly work could examine whether population density cues presented in different sensory modalities (e.g., auditory cues of crowding or heavy traffic; Sng et al., 2017) evoke the same consumption responses as the visual cues used in the present research and whether a multisensory integration of such cues induces a stronger striving for status.

## Conclusion

Thorstein Veblen, a renowned American economist and sociologist, once wrote that *“[t]he basis on which good repute in any highly organized industrial community ultimately rests is pecuniary strength; and the means of showing pecuniary strength, and so of gaining or retaining a good name are leisure and a conspicuous consumption of goods*” (Veblen, 1899, p. 40). In industrialized areas, frequently accompanied by high population density, it appears adaptive for people to flaunt their wealth to achieve a high social standing and reap the benefits that follow from such flashy displays. Our research supports this notion by demonstrating that manipulating perceptions of population density predicts people’s luxury-linked brand attitudes, such that those exposed to high population density cues report more (less) favorable attitudes toward brands deemed to be more (less) luxurious.

## Data Availability Statement

The datasets presented in this study can be found in online repositories. The names of the repository/repositories and accession number(s) can be found below: Data, analysis code, and other replication materials are available through the Open Science Framework (OSF) at https://osf.io/bwje2/.

## Ethics Statement

Ethical review and approval was not required for the study on human participants in accordance with the local legislation and institutional requirements. The patients/participants provided their written informed consent to participate in this study.

## Author Contributions

All authors jointly developed the study design and conceptualization. MF collected and analyzed the data. TO provided funding. All authors drafted and revised the manuscript.

## Conflict of Interest

The authors declare that the research was conducted in the absence of any commercial or financial relationships that could be construed as a potential conflict of interest.

## Publisher’s Note

All claims expressed in this article are solely those of the authors and do not necessarily represent those of their affiliated organizations, or those of the publisher, the editors and the reviewers. Any product that may be evaluated in this article, or claim that may be made by its manufacturer, is not guaranteed or endorsed by the publisher.
