# Supplementary material for: Populated Places and Conspicuous Consumption: High Population Density Cues Predict Consumers’ Luxury-Linked Brand Attitudes
Source: Front Psychol. 2021 Dec 1;12:728903. doi: 10.3389/fpsyg.2021.728903 (PMC8673263; doi:10.3389/fpsyg.2021.728903)
Supplement: Supplementary file 1 [file Data_Sheet_1.pdf]

Appendix: Supplemental Material

Additional Results

Participants in the population density pretest also indicated their affect through the short form of the Positive and Negative Affect Schedule (PANAS; 1 = *very slightly or not at all*; 5 = *extremely*) (Thompson, 2007). We found no significant differences between population density conditions in positive affect ($p > .63$). Participants exposed to high population density cues reported higher negative affect ($p = .032$). Importantly, however, an analysis of covariance (ANCOVA) with population density as the independent variable, negative affect as the covariate, and the manipulation check index as the dependent variable revealed that the effect of population density remained significant ($p = .003$), even after controlling for negative affect, which was not significantly associated with participants' responses on the manipulation check index ($p >.13$). Therefore, our manipulation successfully made people believe that living spaces were scarce (vs. abundant), with negative affect unlikely to be the main driver of this effect.

Data, code, and materials

Raw data, analysis code, and materials used in this research are publicly available through this project's Open Science Framework (OSF) webpage at https://osf.io/bwje2/
